# Impact of Statin Use on Localized Prostate Cancer Outcomes after Radiation Therapy: Long-Term Follow-Up

**DOI:** 10.3390/cancers14153606

**Published:** 2022-07-24

**Authors:** Kevin Kaulanjan, Danny Lavigne, Fred Saad, Pierre I. Karakiewicz, Rocco Simone Flammia, Luis Alex Kluth, Philipp Mandel, Felix K. -H. Chun, Daniel Taussky, Benedikt Hoeh

**Affiliations:** 1 Institut du Cancer de Montréal (ICM), Centre de Recherche du Centre Hospitalier de l’Université de Montréal (CRCHUM), Montréal, QC H2X 0A9, Canada; fredsaad@videotron.ca; 2 Department of Urology, Université des Antilles, CHU de Guadeloupe, 97110 Pointe-à-Pitre, France; 3 Department of Radiation Oncology, Centre hospitalier de l’Université de Montréal, Montréal, QC H2X 0A9, Canada; danny.lavigne@umontreal.ca (D.L.); daniel.taussky.med@ssss.gouv.qc.ca (D.T.); 4 Department of Surgery, Division of Urology, Centre hospitalier de l’Université de Montréal, Montréal, QC H2X 0A9, Canada; pierre.karakiewicz@umontreal.ca; 5 Cancer Prognostics and Health Outcomes Unit, Division of Urology, University of Montréal Health Center, Montréal, QC H2X 0A9, Canada; roccosimone92@gmail.com (R.S.F.); benedikt.hoeh@gmx.de (B.H.); 6 Department of Maternal-Child and Urological Sciences, Sapienza Rome University, Policlinico Umberto I Hospital, 00148 Rome, Italy; 7 Department of Urology, University Hospital Frankfurt, Goethe University Frankfurt am Main, 60318 Frankfurt am Main, Germany; luis.kluth@kgu.de (L.A.K.); philipp.mandel@kgu.de (P.M.); felix.chun@kgu.de (F.K.-H.C.)

**Keywords:** statin, prostate cancer, biochemical recurrence, radiation therapy, brachytherapy

## Abstract

**Simple Summary:**

Statins represent a promising class of agents to improve clinical outcomes of prostate cancer patients treated with radiotherapy, but the results of numerous studies are contradictory. We aimed to assess the impact of statin use on biochemical recurrence in a large database of patients of different risk groups undergoing different modalities of radiation therapy. We evaluated 3555 patients treated with curative external beam radiotherapy, low-dose-rate seed brachytherapy, or external beam radiotherapy plus high-dose-rate brachytherapy. We found no improvement in biochemical recurrence-free survival in statin users, regardless of radiotherapy modality. Our study underlines the need to search for biomarkers that predict an additive effect of statins and determine which patients treated with radiotherapy may benefit from statins as an anticancer drug.

**Abstract:**

The impact of statin use on localized prostate cancer (PCa) remains controversial, especially for patients treated with radiation therapy. We assessed the impact of statin use on biochemical recurrence (BCR) in patients treated for PCa with different modalities of radiation therapy. We evaluated 3555 patients undergoing radiation therapy between January 2001 and January 2022. The impact of statin use on BCR was analyzed for three treatment groups: external beam radiotherapy (EBRT), low-dose-rate seed brachytherapy (LDR), and EBRT plus high-dose-rate brachytherapy (EBRT + HDR). Median follow-up was 52 months among 1208 patients treated with EBRT, 1679 patients treated with LDR, and 599 patients treated with EBRT + HDR. A total of 1544 (43%) patients were taking a statin at the time of treatment, and 497 (14%) patients were in the D’Amico high-risk group. Only intermediate-risk patients treated with LDR fared better with statin use in univariate analysis (*p* = 0.025). This association was not significant in multivariate analysis (HR 0.44, 95% CI 0.18–1.10, *p* = 0.06). Statin use was not associated with a reduced risk of BCR in patients treated with radiation therapy. In the era of precision medicine, further investigation is needed to assess the benefit of statins in well-defined patients.

## 1. Introduction

Radiation therapy is a well-established treatment for all stages of localized prostate cancer (PCa) [1]. However, the management of PCa remains a healthcare challenge, and many patients eventually develop a biochemical recurrence (BCR) following treatment [2]. Among the countless new molecules being studied in the oncology community, multiple lines of evidence indicate that statins may have a benefit in certain cancers [3]. Statins, or 3-hydroxyl-3-methylglutaryl-coenzyme A (HMG-CoA) reductase inhibitors, are a widely used, effective, and well-tolerated medication for hypercholesterolemia. Due to the high prevalence of hypercholesterolemia and PCa among older men [4,5], many PCa patients are likely to have already been prescribed statins at the time of the diagnosis and treatment. Given their antitumor and potentially radiosensitizing properties, statins represent a promising class of agents to improve clinical outcomes of PCa patients treated with radiotherapy [6]. However, in the past 10 years, several meta-analyses showed mixed results regarding statin use to prevent PCa progression [7,8,9,10,11]. One notable study including 489 patients with high-risk cancers found that statin use during radiotherapy was associated with an improved BCR rate [12]. These studies had a limited follow-up, and the impact of statin use on the different modalities of radiation therapy was not explored. Due to differences in the radiobiological effects of low-dose-rate brachytherapy (LDR), high-dose-rate brachytherapy (HDR), and external beam radiotherapy (EBRT), it may be hypothesized that statins have a different radiobiological effect when combined with each technique [13]. Our objective was to assess the impact of statin use on BCR in a large database of patients of different risk groups undergoing different modalities of radiation therapy.

## 2. Materials and Methods

### 2.1. Study Population

The current study was conducted according to the Declaration of Helsinki and approved by the responsible ethical review board (CER CHUM: 21.329). We selected all patients from the prospectively maintained institutional database of the Centre Hospitalier de l’Université de Montréal (CHUM) who underwent radiation therapy as a primary treatment between January 2001 and January 2022. Patients were treated with one of three different modalities of radiation therapy: EBRT, LDR, or EBRT plus high-dose-rate brachytherapy boost (EBRT + HDR). All patients were treated and followed-up in the Department of Radiation Oncology of the CHUM. Risk stratification was performed using National Comprehensive Cancer Network (NCCN) guidelines. Low-risk and favorable intermediate-risk patients were offered either EBRT or LDR brachytherapy. Patients with unfavorable intermediate-risk cancers were offered either EBRT or a combination of EBRT with HDR brachytherapy depending on life expectancy and comorbidity. Androgen deprivation therapy was added for typically 6–36 months for all patients presenting with unfavorable intermediate-risk and all patients with high-risk cancers. Final decisions were taken with shared decision-making. Typical follow-up was every 3–4 months post treatment for the first 2 years, followed by every 6 months up to 5 years. Following the 5-year mark, follow-up was every 8–12 months. Patients who had received radiotherapy for reasons other than a primary curative-intent treatment were excluded from further statistical considerations. Patients with missing clinical parameters required for D’Amico risk classification (*n* = 50) or with missing follow-up data (*n* = 278) were initially included and tabulated but excluded from further BCR-free survival analyses.

### 2.2. Study Variables and Outcomes

Data regarding tumor characteristics at diagnosis included prostate-specific antigen (PSA), clinical T-stage, and Gleason score. Statin use was defined as statin intake at the time of radiation therapy treatment. The modality of radiation treatment (EBRT, LDR, or EBRT + HDR) and the use of androgen deprivation therapy (ADT) were collected. Each patient was stratified into low, intermediate, or high risk according to the D’Amico classification. Low risk was defined as Gleason score ≤ 6, PSA < 10 ng/mL, and clinical T-stage ≤ T2a. Intermediate risk was defined as Gleason score = 7, PSA = 10–20 ng/mL, and/or clinical T-stage = T2b. High risk was defined as any one of the following features: Gleason score ≥ 8, PSA > 20, or clinical T-stage ≥ T2c [14,15]. BCR was based on the Phoenix criteria, defined as a PSA rise of at least 2 ng/mL above the nadir value following irradiation [16].

### 2.3. Statistical Analysis

Descriptive statistics were reported as medians and interquartile ranges (IQRs) for continuous variables, and as frequencies and percentages for categorical variables. The chi-squared test was used to analyze categorical variables, while the Kruskal–Wallis test was used for quantitative variables. Statistical analyses consisted of five steps. First, patient and clinicopathological characteristics were tabulated according to statin use within the overall cohort, irrespective of D’Amico risk stratification or treatment modality. Thereafter, Kaplan–Meier plots tested for differences in BCR-free survival rates according to statin use. Third, univariable and multivariable Cox regression models tested for an independent predictor status of statin use on BCR. Here, adjustment variables consisted of D’Amico risk classification (low vs. intermediate vs. high), radiotherapy modality (LDR vs. EBRT vs. EBRT + HDR), age (continuously coded), PSA (0–10 vs. >10–20 vs. >20 ng/mL), and ADT (no vs. yes). Fourth, statistical analyses were repeated in EBRT-treated patients solely. Specifically, analyses were refitted in D’Amico intermediate- and high-risk EBRT-treated patients. Here, adjustment variables in Cox regression models consisted of age (continuously coded), PSA (0–10 vs. >10–20 vs. >20 ng/mL), and ADT (no vs. yes). Fifth, statistical analyses were repeated on LDR-treated patients solely. Specifically, analyses were refitted in D’Amico low- and intermediate-risk LDR-treated patients. Here, adjustment variables in Cox regression models consisted of age (continuously coded), ADT (no vs. yes), and PSA (0–10 vs. >10–20 (for intermediate D’Amico risk)). Statistical analyses were performed using R Version 3.4.3 (The R foundation). All tests were two-sided at a level of significance of *p* < 0.05.

## 3. Results

### 3.1. Descriptive Characteristics of the Study Population

A total of 3555 PCa patients represented the population of the current study. Among those, 1544 (43%) patients were treated with statins, whereas 2011 (57%) were nonusers. EBRT, LDR, and EBRT + HDR was administered to 1208 (35%), 1679 (48%), and 599 (17%) patients, respectively. Following D’Amico risk stratification, 1154 (33%), 1854 (53%), and 497 (14%) qualified for low-, intermediate-, and high-risk classification, respectively (Table 1).

### 3.2. Biochemical Recurrence within the Overall Study Population According to Statin Use

**Figure 1:**
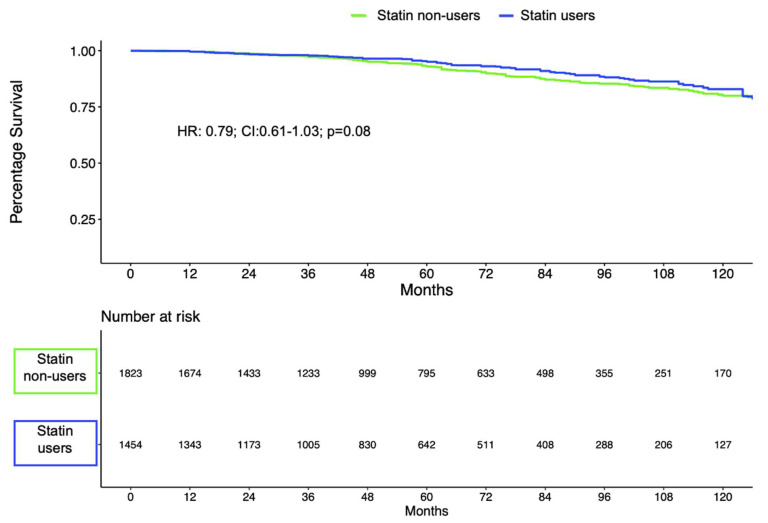
BCR-free survival estimated by Kaplan–Meier and stratified by statin intake.

Of 3555 patients, 3277 (92%) had complete data and were eligible for survival analyses, of which 1454 (44%) were treated with statins. BCR-free survival rates at 5 and 10 years for patients treated with statins vs. patients with no statins were 95% vs. 93% and 83% vs. 80%, respectively (log-rank = 0.1; Figure 1). Statin use failed to reach statistical significance as an independent predictor of BCR in multivariable Cox regression models after adjustment for covariables (hazard ratio (HR): 0.79; 95% CI: 0.61–1.03; *p* = 0.08; Table 2).

### 3.3. Biochemical Recurrence within EBRT-Treated Patients According to Statin Use

**Figure 2:**
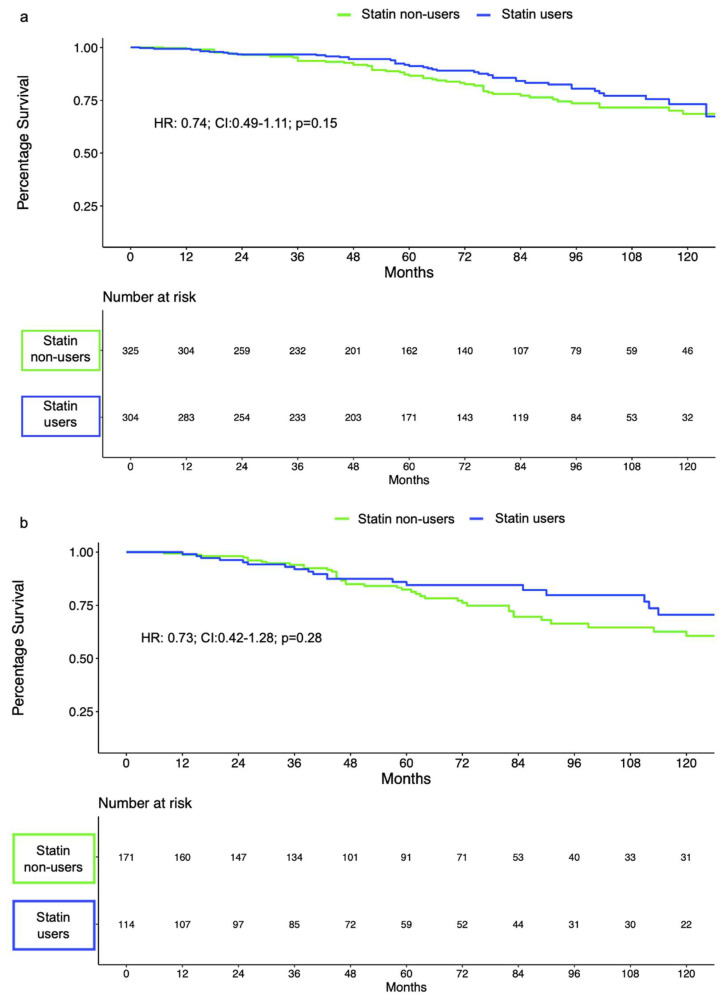
BCR-free survival in D’Amico intermediate-risk (**a**) and high-risk (**b**) patients treated with EBRT.

Of 1208 EBRT-treated patients, 645 (54%) and 302 (25%) were intermediate- and high-risk according to the D’Amico risk classification (Tables S1–S3 and S6). Of those, 309 (48%) intermediate- and 122 (40%) high-risk patients were treated with statins. In intermediate-risk EBRT-treated patients, BCR-free survival rates at 5 and 10 years for statin users vs. nonusers were 91% vs. 87% and 73% vs. 69%, respectively. In high-risk EBRT-treated patients, 5 year and 10 year BCR-free survival rates were 85% vs. 82% and 71% vs. 61% for statin users vs. nonusers, respectively. BCR-free survival rates did not differ according to statin use (log-rank > 0.2; Figure 2a,b). Statin use failed to reach statistical significance as an independent predictor of BCR in separate multivariable Cox regression models after adjustment for covariables in both intermediate- (HR: 0.74; 95% CI: 0.49–1.11; *p* = 0.15) and high-risk EBRT-treated patients (HR: 0.73; 95% CI: 0.42–1.28; *p* = 0.28; Table 3).

### 3.4. Biochemical Recurrence within LDR-Treated Patients According to Statin Use

**Figure 3:**
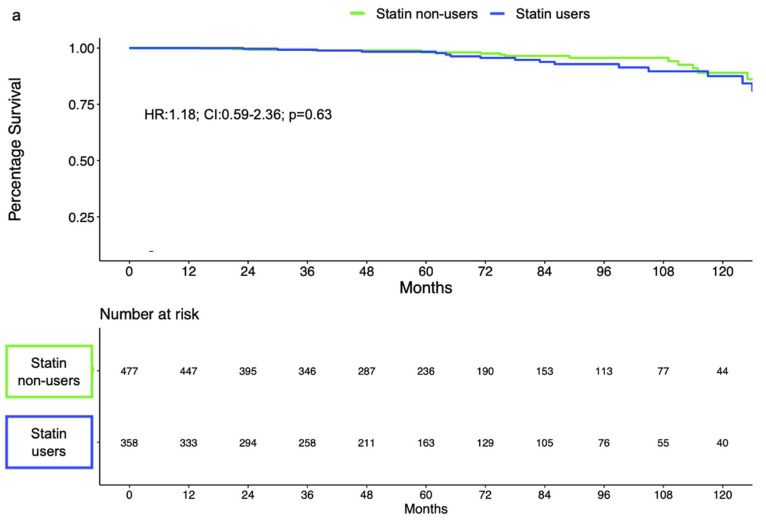

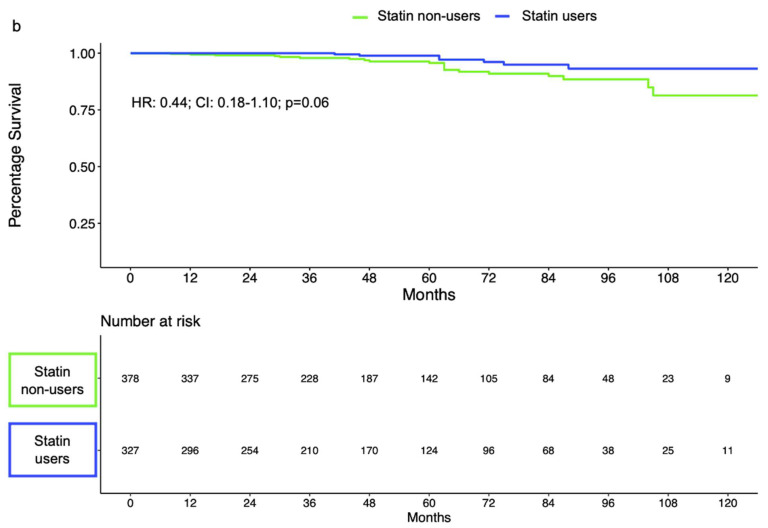
BCR-free survival in D’Amico low-risk (**a**) and intermediate-risk (**b**) patients treated with LDR.

Of 1679 LDR-treated patients, 885 (53%) and 779 (46%) were low- and intermediate-risk according to the D’Amico risk classification, respectively (Tables S4 and S5). Of those, 379 (43%) low- and 356 (46%) intermediate-risk patients were treated with statins. In low-risk LDR-treated patients, BCR-free survival rates at 5 and 10 years were 98% vs. 98% and 87% vs. 89% for statin users vs. nonusers, respectively. In intermediate-risk LDR-treated patients, 5 year and 10 year BCR-free survival rates were 99% vs. 96% and 93% vs. 81%, respectively. BCR-free survival rates did not differ according to statin use in low-risk LDR-treated patients (log-rank = 0.48; Figure 3a). By contrast, in intermediate-risk patients, statin users demonstrated more favorable BCR-free survival rates (log-rank = 0.025; Figure 3b). However, statin use failed to reach statistical significance as an independent predictor of BCR in separate multivariable Cox regression models after adjustment for covariables in both low- (HR:1.18; 95% CI: 0.59–2.36; *p* = 0.63) and intermediate-risk LDR-treated patients (HR: 0.44; 95% CI: 0.18–1.10; *p* = 0.06; Table 4).

## 4. Discussion

Among our 3555 patients treated with external beam radiotherapy, low-dose-rate seed brachytherapy, or EBRT plus high-dose-rate brachytherapy boost, statin use was not associated with an improved BCR rate, regardless of radiotherapy modality. We found a trend in favor of statin users for intermediate-risk EBRT-treated patients; BCR-free survival rates at 5 and 10 years were higher for statin users vs. nonusers (91% vs. 87% and 73% vs. 69%, respectively). Likewise, in high-risk EBRT-treated patients, 5 year and 10 year BCR-free survival rates were in favor of statin users vs. nonusers (85% vs. 82% and 71% vs. 61%, respectively). The same trend was found in intermediate-risk LDR-treated patients: 5 year and 10 year BCR-free survival rates were 99% vs. 96% and 93% vs. 81%, respectively. However, only intermediate-risk patients treated with LDR fared better with statin use in univariate analysis (*p* = 0.025). This association was not significant in multivariate analysis (HR 0.44, 95% CI 0.18–1.10, *p* = 0.06). Although several studies found no effect of statin use on biochemical progression after radical prostatectomy, results after radiation therapy are mixed. Our results are consistent with several studies reporting no BCR benefit from the combination of statin with EBRT or brachytherapy [17,18,19,20,21]. However, in contrast to surgical series, some radiation studies did find an improvement in BCR-free survival in patients treated with statins. One notable example is Oh et al., who reported an association between statin use and improvement in freedom from biochemical failure in a cohort of PCa patients treated with brachytherapy [22]. Furthermore, Gutt et al. and Kollmeier et al. found a significant reduction in the risk of BCR after EBRT, particularly in high-risk patients [12,23]. In a recent meta-analysis, Yin et al. pooled all these previous studies and found that statin use after a curative treatment did not improve BCR-free survival overall (*p* = 0.33), whereas it did improve BCR-free survival in high-risk patients (*p* < 0.01) [7]. With a slightly larger population than Kollmeier et al., we did not find such an effect in our 497 high-risk patients. Of note, only 23% of their overall population and 16% of their high-risk patients were statin users, compared to 39% in our high-risk cohort.

We found that the lowest percentage of statin users was found in the high-risk group (39%) compared to 41% in the low-risk group and 47% in the intermediate-risk group. The possible influence of statin use on high-risk cancers may be due to its direct influence on androgen production, which could result in reducing the incidence of high-risk cancers. Statins are competitive inhibitors of 3-hydroxyl-3-methylglutaryl-coenzyme A (HMG-CoA) reductase. By blocking the active site of the enzyme, statins inhibit the conversion of HMG-CoA to mevalonate. Mevalonate is the proximal step of cholesterol biosynthesis. As a result, statins deplete intracellular cholesterol, which is a precursor for the biosynthesis of all steroid hormones, including androgens. Consistent with this hypothesis, statins have been shown to inhibit androgen receptor (AR) activity in PCa cell lines [3].

In addition to the lipid-lowering effect, statins have pleiotropic properties such as tumor growth inhibition and apoptosis induction. Among these properties, statins seem to have a radiosensitizing effect. In vitro, HMG-CoA reductase inhibitors induce a late arrest in the G1 phase of the cell cycle, during which the cells are known to be more sensitive to radiation-induced cell death. Statins might also contribute to a radiosensitizing effect in PCa through several other mechanisms, including the EGFR–Ras–ERK1/2 pathway [24].

Although our data were collected from a prospectively maintained database, our study must be interpreted in light of its retrospective nature. The major limitation of our study includes the lack of details regarding the duration and dosage of statin treatments. Several prior studies have demonstrated that the antineoplastic effect of statins is dose-dependent [12,25]. The dose could potentially play a role in the radiosensitizing effect. The type of statin medication used was also unknown. However, there is evidence supporting that hydrophobic statins (such as simvastatin or lovastatin) have a better antitumoral effect than hydrophilic statins [26]. Thus, more well-designed prospective studies are needed to provide a more robust conclusion. Unfortunately, we have no data about race or socioeconomic differences that could have influenced our results in our database. Moreover, with our follow-up, we had only a few deceased patients in our database, which prevented us from conducting analyses regarding overall survival events. Despite these limitations, our study showed the impact of statin use on contemporary oncological outcomes of PCa in a large cohort of patients treated with several modalities of radiation therapy with a long-term follow-up.

## 5. Conclusions

Statin use was not associated with a reduced risk of BCR in patients treated with different radiotherapy modalities for localized PCa. In the era of precision medicine, searching for biomarkers that predict an additive effect of statins for patients treated with radiotherapy or focusing on patients with high-risk cancers could be the key to the success of statins as an anticancer drug.

## Figures and Tables

**Table 1:** Patient and clinicopathological characteristics tabulated according to statin use. All values are medians (IQR) or frequencies (%).

	*n*	Overall *n* = 3555	No Statin *n* = 2011 (57%)	Statin Intake *n* = 1544 (43%)	*p*-Value
Age (years)	3520	67 (62, 72)	66 (61, 71)	68 (64, 72)	<0.001
PSA (ng/mL)	3521				<0.001
0–10		2773 (79%)	1507 (76%)	1266 (82%)	
10–20		564 (16%)	343 (17%)	221 (14%)	
>20		184 (5.2%)	128 (6.5%)	56 (3.6%)	
Gleason score	3479				0.080
6		1417 (41%)	827 (42%)	590 (39%)	
7		1764 (51%)	949 (49%)	815 (53%)	
8		199 (5.7%)	116 (6.0%)	83 (5.4%)	
9		91 (2.6%)	52 (2.7%)	39 (2.5%)	
10		8 (0.2%)	3 (0.2%)	5 (0.3%)	
Clinical T Stage	3498				0.046
cT1		2211 (63%)	1253 (64%)	958 (62%)	
cT2a		884 (25%)	477 (24%)	407 (26%)	
cT2b		176 (5.0%)	89 (4.5%)	87 (5.7%)	
cT2c		47 (1.3%)	34 (1.7%)	13 (0.8%)	
cT3-4		180 (5.1%)	106 (5.4%)	74 (4.8%)	
Treatment	3486				0.9
EBRT		1208 (35%)	686 (35%)	522 (34%)	
LDR		1679 (48%)	941 (48%)	738 (48%)	
EBRT + HDR		599 (17%)	333 (17%)	266 (17%)	
ADT	3555				0.5
No		2991 (84%)	1685 (84%)	1306 (85%)	
Yes		564 (16%)	326 (16%)	238 (15%)	
D’Amico Classification	3505				<0.001
Low-risk		1154 (33%)	677 (34%)	477 (31%)	
Intermediate-risk		1854 (53%)	984 (50%)	870 (56%)	
High-risk		497 (14%)	302 (15%)	195 (13%)	
Follow-up (months)	3277	52 (28, 84)	51 (24, 84)	52 (30, 84)	0.4

PSA: prostate-specific antigen, EBRT: external beam radiation therapy, LDR: low-dose-rate brachytherapy, HDR: high-dose-rate brachytherapy, ADT: androgen deprivation therapy.

**Table 2:** Univariable and multivariable Cox regression models predicting biochemical recurrence within the overall study population, stratified according to statin use.

	Univariable			Multivariable ^1^		
	Hazard Ratio	95% CI	*p*-Value	Hazard Ratio	95% CI	*p*-Value
Statin treatment						
No	Ref.			Ref.		
Yes	0.80	0.62–1.04	0.10	0.79	0.61–1.03	0.08

^1^ Adjustment covariables consisted of D’Amico risk classification, radiotherapy modality, age, PSA, and ADT. PSA: prostate-specific antigen; ADT: androgen deprivation therapy; 95% CI: 95% confidence interval; Ref.: reference.

**Table 3:** Univariable and multivariable Cox regression models predicting biochemical recurrence among EBRT-treated patients (intermediate- and high-risk), according to statin use.

		Univariable			Multivariable ^1^		
		Hazard Ratio	95% CI	*p*-Value	Hazard Ratio	95% CI	*p*-Value
D’Amico **intermediate**-risk	Statin treatment						
	No	Ref.			Ref.		
	Yes	0.79	0.52–1.17	0.24	0.74	0.49–1.11	0.15
D’Amico **high**-risk	Statin treatment						
	No	Ref.			Ref.		
	Yes	0.70	0.41–1.19	0.19	0.73	0.42–1.28	0.28

^1^ Adjustment covariables consisted of age, PSA, and ADT. PSA: prostate-specific antigen; ADT: androgen deprivation therapy; 95% CI: 95% confidence interval; Ref.: reference.

**Table 4:** Univariable and multivariable Cox regression models predicting biochemical recurrence among LDR-treated patients (low and intermediate), according to statin use.

		Univariable			Multivariable ^1^		
		Hazard Ratio	95% CI	*p*-Value	Hazard Ratio	95% CI	*p*-Value
D’Amico **low**-risk	Statin treatment						
	No	Ref.			Ref.		
	Yes	1.27	0.64–2.52	0.48	1.18	0.59–2.36	0.63
D’Amico **intermediate**-risk	Statin treatment						
	No	Ref.			Ref.		
	Yes	0.38	0.16–0.92	0.03	0.44	0.18–1.10	0.06

^1^ Adjustment covariables consisted of age, PSA, and ADT. PSA: prostate-specific antigen; ADT: androgen deprivation therapy; 95% CI: 95% confidence interval; Ref.: reference.

## Data Availability

Data presented are contained within the article; for additional information, datasets are also available upon request from the corresponding author.

## Supplementary Materials

The following supporting information can be downloaded at https://www.mdpi.com/article/10.3390/cancers14153606/s1: Table S1. Characteristics of D’Amico low-risk EBRT-treated patients according to statin use; Table S2. Characteristics of D’Amico intermediate-risk EBRT-treated patients according to statin use; Table S3. Characteristics of D’Amico high-risk EBRT-treated patients according to statin use; Table S4. Characteristics of D’Amico low-risk LDR-treated patients according to statin use; Table S5. Characteristics of D’Amico intermediate-risk LDR-treated patients according to statin use; Table S6. Characteristics of patients treated by EBRT+ HDR according to statin use.
