# Supplementary material for: Impact of Statin Use on Localized Prostate Cancer Outcomes after Radiation Therapy: Long-Term Follow-Up
Source: Cancers (Basel). 2022 Jul 24;14(15):3606. doi: 10.3390/cancers14153606 (PMC9331711; doi:10.3390/cancers14153606)
Supplement: Supplementary file 1 [file cancers-14-03606-s001.zip › cancers-1803025-supplementary.pdf]

Supplementary Data

Supplementary Table S1: Characteristics of D'Amico Low-risk EBRT-treated patients according to statin use. All values are median (IQR) or frequencies (%).

	N	Overall, N = 254	No Statin, N = 163 (64%)	Statin Intake, N = 91 (36%)	p-value
**Age (years)**	253	68.0 (64.0, 72.0)	68.0 (63.0, 72.0)	69.0 (65.0, 72.8)	0.090
**Follow-up (months)**	239	90 (56, 111)	84 (48, 108)	96 (64, 114)	0.2
**PSA (ng/mL)**	254				>0.9
0-10		254 (100%)	163 (100%)	91 (100%)	
10-20		0 (0%)	0 (0%)	0 (0%)	
>20		0 (0%)	0 (0%)	0 (0%)	
**Gleason Score**	254				>0.9
6		254 (100%)	163 (100%)	91 (100%)	
**Clinical T-Stage**	254				0.3
cT1		182 (72%)	113 (69%)	69 (76%)	
cT2a		72 (28%)	50 (31%)	22 (24%)	
**ADT**	254				
No		254 (100%)	163 (100%)	91 (100%)	

Abbreviations: PSA=Prostate-specific antigen; ADT=Androgen deprivation therapy

Supplementary Table S2. Characteristics of D'Amico intermediate-risk EBRT-treated patients according to statin use; All values are median (IQR) or frequencies (%).

	N	Overall, N = 645	No Statin, N = 336 (52%)	Statin Intake, N = 309 (48%)	p-value
**Age (years)**	643	71.0 (66.0, 74.0)	71.0 (65.0, 74.0)	71.0 (66.0, 74.0)	0.4
**Follow-up (month)**	629	60 (30, 96)	59 (30, 91)	66 (36, 96)	0.2
**PSA (ng/mL)**	645				0.001
0-10		448 (69%)	214 (64%)	234 (76%)	
10-20		197 (31%)	122 (36%)	75 (24%)	
>20		0 (0%)	0 (0%)	0 (0%)	
**Gleason Score**	631				0.11
6		80 (13%)	48 (15%)	32 (10%)	
7		551 (87%)	278 (85%)	273 (90%)	
**Clinical T-stage**	641				0.3
cT1		392 (61%)	205 (61%)	187 (61%)	
cT2a		188 (29%)	103 (31%)	85 (28%)	
cT2b		61 (9.5%)	26 (7.8%)	35 (11%)	
**ADT**	645				0.15
No		550 (85%)	280 (83%)	270 (87%)	
Yes		95 (15%)	56 (17%)	39 (13%)	

Abbreviations: PSA=Prostate-specific antigen; ADT=Androgen deprivation therapy; EBRT=External beam radiation therapy

Supplementary Table S3: Characteristics of D'Amico high-risk EBRT-treated patients according to statin use; All values are median (IQR) or frequencies (%).

	**N**	**Overall, N = 302**	**No Statin, N = 180 (60%)**	**Statin Intake, N = 122 (40%)**	**p-value**
**Age (years)**	298	71 (66, 75)	71 (66, 75)	72 (66, 74)	0.8
**Follow-up (months)**	285	60 (36, 91)	60 (36, 90)	60 (34, 108)	0.8
**PSA (ng/mL)**	301				0.008
0-10		116 (39%)	59 (33%)	57 (47%)	
10-20		67 (22%)	38 (21%)	29 (24%)	
>20		118 (39%)	83 (46%)	35 (29%)	
**Clinical T-stage**	302				0.12
cT1		78 (26%)	45 (25%)	33 (27%)	
cT2a		54 (18%)	38 (21%)	16 (13%)	
cT2b		26 (8.6%)	18 (10%)	8 (6.6%)	
cT2c		25 (8.3%)	17 (9.4%)	8 (6.6%)	
cT3-4		119 (39%)	62 (34%)	57 (47%)	
**Gleason Score**	299				0.5
6		38 (13%)	24 (13%)	14 (12%)	
7		85 (28%)	55 (31%)	30 (25%)	
8		112 (37%)	65 (37%)	47 (39%)	
9		60 (20%)	33 (19%)	27 (22%)	
10		4 (1.3%)	1 (0.6%)	3 (2.5%)	
**ADT**	302				0.2
No		36 (12%)	25 (14%)	11 (9.0%)	
Yes		266 (88%)	155 (86%)	111 (91%)	

Abbreviations: PSA=Prostate-specific antigen; ADT=Androgen deprivation therapy; EBRT=External beam radiation therapy

Supplementary Table S4. Characteristics of D'Amico low-risk LDR-treated patients according to statin use; All values are median (IQR) or frequencies (%).

	N	Overall, N = 885	No Statin, N = 506 (57%)	Statin Intake, N = 379 (43%)	p-value
**Age (years)**	885	64 (59, 68)	63 (58, 68)	66 (61, 69)	<0.001
**Follow-up (months)**	835	56 (30, 90)	56 (30, 90)	52 (30, 86)	0.5
**PSA (ng/mL)**	885				>0.9
0-10		885 (100%)	506 (100%)	379 (100%)	
10-20		0 (0%)	0 (0%)	0 (0%)	
>20		0 (0%)	0 (0%)	0 (0%)	
**Clinical T-stage**	885				0.090
cT1		674 (76%)	396 (78%)	278 (73%)	
cT2a		211 (24%)	110 (22%)	101 (27%)	
**Gleason Score**	885				>0.9
6		885 (100%)	506 (100%)	379 (100%)	
**ADT**	885				>0.9
No		882 (100%)	504 (100%)	378 (100%)	
Yes		3 (0.3%)	2 (0.4%)	1 (0.3%)	

Abbreviations: PSA=Prostate-specific antigen; ADT=Androgen deprivation therapy; LDR=Low-dose rate seed brachytherapy

Supplementary Table S5. Characteristics of D'Amico intermediate-risk LDR-treated patients according to statin use; All values are median (IQR) or frequencies (%).

	N	Overall, N = 779	No Statin, N = 423 (54%)	Statin Intake, N = 356 (46%)	p-value
**Age (years)**	777	66 (62, 70)	65 (60, 70)	67 (63, 71)	<0.001
**Follow-up (months)**	705	48 (24, 72)	44 (22, 72)	48 (24, 72)	0.4
**PSA (ng/mL)**	779				0.11
0-10		662 (85%)	351 (83%)	311 (87%)	
10-20		117 (15%)	72 (17%)	45 (13%)	
>20		0 (0%)	0 (0%)	0 (0%)	
**Clinical T-stage**	775				0.5
cT1		577 (74%)	320 (76%)	257 (72%)	
cT2a		158 (20%)	80 (19%)	78 (22%)	
cT2b		40 (5.2%)	20 (4.8%)	20 (5.6%)	
**Gleason Score**	768				0.8
6		121 (16%)	67 (16%)	54 (15%)	
7		647 (84%)	350 (84%)	297 (85%)	
**ADT**	779				0.3
No		772 (99%)	421 (100%)	351 (99%)	
Yes		7 (0.9%)	2 (0.5%)	5 (1.4%)	

Abbreviations: PSA=Prostate-specific antigen; ADT=Androgen deprivation therapy; LDR=Low-dose rate seed brachytherapy

Supplementary Table S6. Characteristics of patients treated by EBRT+HDR according to statin use; All values are median (IQR) or frequencies (%).

	N	Overall, N = 599	No Statin, N = 333 (56%)	Statin Intake, N = 266 (44%)	p-value
**Age (years)**	596	67 (62, 71)	66 (61, 70)	68 (64, 72)	<0.001
**Follow-up (months)**	539	36 (18, 52)	36 (18, 54)	36 (22, 52)	0.7
**PSA (ng/mL)**	599				0.010
0-10		372 (62%)	191 (57%)	181 (68%)	
10-20		166 (28%)	99 (30%)	67 (25%)	
>20		61 (10%)	43 (13%)	18 (6.8%)	
**Gleason Score**	598				0.6
6		29 (4.8%)	13 (3.9%)	16 (6.0%)	
7		454 (76%)	252 (76%)	202 (76%)	
8		84 (14%)	48 (14%)	36 (14%)	
9		28 (4.7%)	18 (5.4%)	10 (3.8%)	
10		3 (0.5%)	1 (0.3%)	2 (0.8%)	
**Clinical T-stage**	598				0.002
cT1		282 (47%)	162 (49%)	120 (45%)	
cT2a		189 (32%)	88 (27%)	101 (38%)	
cT2b		49 (8.2%)	25 (7.5%)	24 (9.0%)	
cT2c		21 (3.5%)	16 (4.8%)	5 (1.9%)	
cT3-4		57 (9.5%)	41 (12%)	16 (6.0%)	
**D'Amico Classification**	599				0.055
Low risk		9 (1.5%)	4 (1.2%)	5 (1.9%)	
Intermediate risk		408 (68%)	215 (65%)	193 (73%)	
High risk		182 (30%)	114 (34%)	68 (26%)	
**ADT**	599				0.7
No		420 (70%)	231 (69%)	189 (71%)	
Yes		179 (30%)	102 (31%)	77 (29%)	

Abbreviations: PSA=Prostate-specific antigen; ADT=Androgen deprivation therapy; EBRT=External beam radiation therapy.
